# Supplementary material for: Modifying muscle metabolic dysregulation in inclusion body myositis with pioglitazone: a single-arm trial
Source: Nat Commun. 2026 Mar 15;17:3995. doi: 10.1038/s41467-026-70262-0 (PMC13136323; doi:10.1038/s41467-026-70262-0)
Supplement: Supplementary file 1 — Supplementary information [file 41467_2026_70262_MOESM1_ESM.pdf]

Supplemental Figures:

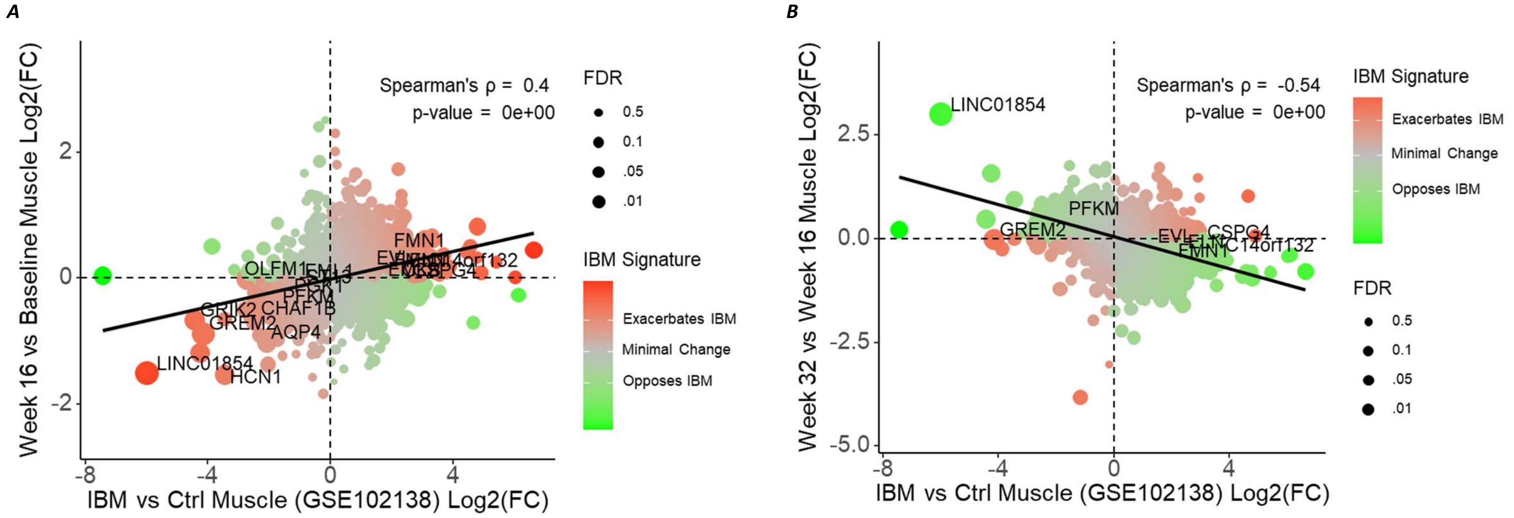

Supplementary Figure 1. *Correlation analysis comparing the transcriptomic signature in muscle from 13 patients with inclusion body myositis (IBM) during the A) lead-in period off therapy (baseline to week 16) and B) during the treatment period on pioglitazone (week 16 to week 32). The transcriptomic disease signature of IBM was derived from a publicly available transcriptomic dataset (GSE102138) in which IBM and control skeletal muscle samples were compared. Metabolites in green indicate a shift away from the IBM transcriptomic signature, while metabolites in red indicate a shift in the same direction as the IBM signature. The significance of the correlation was determined using Spearman's correlation coefficient, with FDR representing Benjamini-Hochberg corrected Fisher's combined p-values across both comparisons (FDR < 0.05 labeled).*

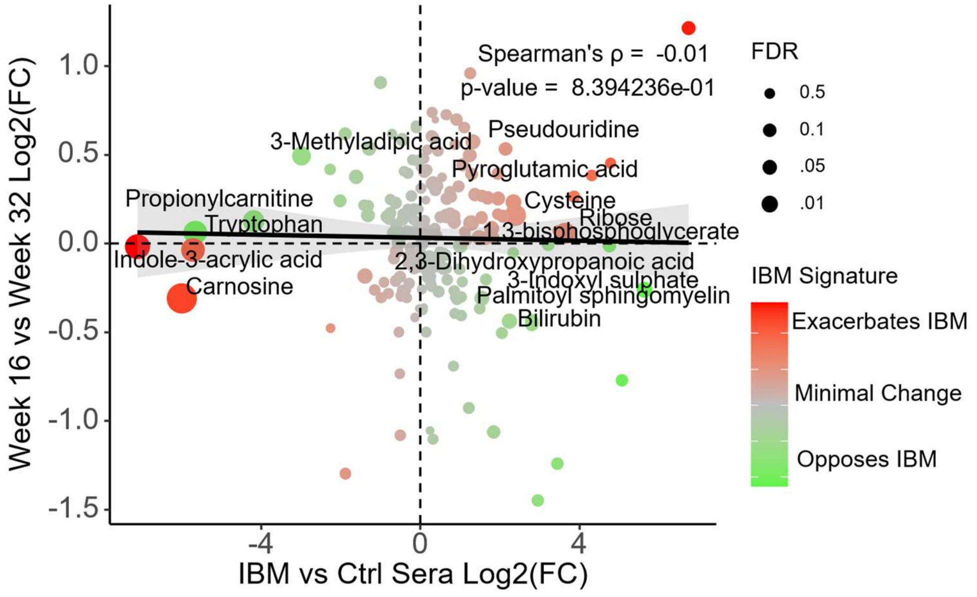

Supplementary Figure 2*. Correlation analyses comparing the metabolic signature in sera from 13 patients with inclusion body myositis (IBM) at the end of the intervention period with pioglitazone (week 32) versus week 16. Metabolites in green indicate a shift away from the IBM metabolic signature, while metabolites in red indicate a shift in the same direction as the IBM signature. The significance of the correlation was determined using Spearman's correlation coefficient, with FDR representing Benjamini-Hochberg corrected Fisher's combined p-values across both comparisons (FDR < 0.05 labeled).*

Supplemental Tables:

**Baseline characteristics**	**IBM (n=16)**
Age (mean years ± SD)	66.8 ± 7.3
Female (n (%))	8/16 (50%)
Caucasian (n (%))	16/16 (100%)
Disease duration (mean years ± SD)	5.4 ± 4.4
Mean CK (mean ± SD)	482.8 ± 387.4
Use of assistive device	4/16 (25%)
+ CN1a antibody	6/14 (42%)
Mitochondrial changes on biopsy (COX negative fibers)	12/16 (75%)
Primary inflammation on biopsy	15/16 (93%)
Rimmed vacuoles on biopsy	10/16 (62%)

***Supplementary Table 1**. Demographics and clinical features of the enrolled inclusion body myositis (IBM) study population*

**Demographics**	**IBM**	**Control muscle**		**Control sera**	
Age (mean years ± SD)	66.8 ± 7.3	60.6 ± 15.4	P=0.20	56.3 ± 5.7 **	P=0.001
Female (n (%))	8/16 (50%)	4/5 (80%)	P=0.34	7/10 (70%)	P=0.43
Caucasian (n (%))	16/16 (100%)	5/5 (100%)	P=1.0	4/10 (40%) *	P=0.01

***Supplementary Table 2.** Demographics of the enrolled inclusion body myositis (IBM) study population (n=16), healthy control sera (n=10) and healthy control muscle (n=5). Comparisons between IBM vs. control muscle and IBM vs. control sera were made using the Wilcoxon rank-sum test and the Fisher's exact test all two-sided. * p< 0.05, ** p< 0.01.*

Supplementary Table 3*. Comprehensive list of metabolites showing differential abundance in muscle (FDR < 0.05) between inclusion body myositis patients (n=15) and healthy controls (n=5). Determined using limma ebayes function with adjustment for multiple comparisons.*

Compound	log2FC	t	P.Value	FDR
Propionylcarnitine	-6.927	-6.14516	2.63E-06	0.000558
trans-3-Indoleacrylic acid	-4.4376	-5.03968	4.00E-05	0.003193
Tryptophan	-4.185	-4.89796	5.70E-05	0.003193
CMP	-2.6258	-4.87616	6.03E-05	0.003193
UMP	-3.2572	-4.70137	9.35E-05	0.00347
Tartaric acid	3.5018	4.681822	9.82E-05	0.00347
Phenylacetylglutamine	2.8234	4.399223	0.0002	0.006058
Glucose 1-phosphate	-2.78	-4.16405	0.000361	0.008847
Mannose 6-phosphate	-2.9011	-4.14872	0.000376	0.008847
Piperine	4.7904	3.879393	0.000737	0.01563
reduced Glutathione	4.5905	3.837856	0.000818	0.01576
Cysteinylglycine	2.955	3.801785	0.000895	0.015804
1-Methylguanine	1.68	3.644432	0.001322	0.021556
Ursolic acid	-3.652	-3.59628	0.001489	0.022545
3-Indoxyl sulphate	2.1566	3.523211	0.001782	0.02519
Methylparaben	3.9029	3.462985	0.002066	0.026204
GMP	-2.2045	-3.4561	0.002101	0.026204
Cytosine	-3.0872	-3.26738	0.003326	0.03673
Methylguanosine	2.1541	3.238498	0.003566	0.03673
Vanillin	3.1603	3.216947	0.003756	0.03673
Argininosuccinic acid	-2.0191	-3.1993	0.003919	0.03673
Adenosine	3.0938	3.172628	0.004178	0.03673
Testosterone sulfate	2.9795	3.158329	0.004324	0.03673
Lactic acid	-1.8878	-3.14588	0.004455	0.03673
Maltol	-1.7442	-3.14531	0.004461	0.03673
Cytidine	-3.0627	-3.14124	0.004505	0.03673
cis-Aconitic acid	4.9328	3.114445	0.004803	0.037709
Cysteinesulfinic acid	-2.4288	-3.07996	0.005214	0.038162
Cysteine-S-sulfate	6.493	3.079439	0.00522	0.038162
trans,trans-2,4-Heptadienal	2.6412	3.059099	0.005479	0.038718
Uracil	3.7148	3.040578	0.005725	0.039152
5-Methoxysalicylic acid	1.2075	2.980953	0.006591	0.043669

Supplementary Table 4*. Comprehensive list of all detected metabolites in KEGG pathways for glutathione metabolism, glycolysis, TCA cycle, and oxidative phosphorylation, comparing muscle samples from inclusion body myositis patients (n=15) to control muscle samples (n=5). Log2 fold change (Log2FC) and p-values are provided for each metabolite. Determined using limma ebayes function with adjustments for multiple comparison.*

Compound	log2FC	t	P.Value	FDR
Glucose 1-phosphate	-2.78	-4.16405	0.000361	0.008847
reduced Glutathione	4.5905	3.837856	0.000818	0.01576
Cysteinylglycine	2.955	3.801785	0.000895	0.015804
Lactic acid	-1.8878	-3.14588	0.004455	0.03673
cis-Aconitic acid	4.9328	3.114445	0.004803	0.037709
Citric acid	7.6544	2.454945	0.021905	0.098807
Cysteine	3.8033	2.385669	0.025489	0.101954
oxidized Glutathione	-1.4787	-2.31235	0.029863	0.109153
Fumarate	-1.2781	-2.03592	0.053199	0.155909
ADP	-0.85403	-2.02497	0.054392	0.155909
Malic acid	-1.2656	-2.02471	0.054421	0.155909
Fructose 1,6 Bisphosphate	-0.71111	-1.93483	0.065154	0.174844
1,3-bisphosphoglycerate	5.0345	1.623317	0.11788	0.268716
Glutamate	1.0354	1.580162	0.127456	0.278563
Succinate	-1.4921	-1.55818	0.132576	0.283901
Alpha-ketoglutaric acid	2.5953	1.434956	0.164494	0.324567
Nicotinamide adenine dinucleotide (NAD+)	2.1716	1.292151	0.208885	0.379151
ATP	5.9195	0.955896	0.348869	0.52674
Dihydroxyacetone Phosphate(glycerone phosphate)	0.095842	-0.95294	0.350331	0.52674
Glucose	0.15844	-0.67618	0.505537	0.653298
Glycine	0.3759	0.58972	0.561014	0.687486
Phosphoenolpyruvate	1.1277	0.562061	0.579404	0.699163
Glucose 6-phosphate	3.4486	0.552341	0.585937	0.701801
3-Phosphoglyceric acid	-0.37277	-0.48201	0.634264	0.747022
Pyruvic acid	0.79354	-0.43575	0.667001	0.7727
Pyroglutamic acid	-0.07655	0.112317	0.911527	0.966219

Supplementary Table 5. *Muscle ultrasound severity score and IBM-Functional Rating Scale (IBM-FRS) for each of the study participants at the baseline visit.*

**Patient**	**US score**	**Atrophy present**	**IBM-FRS**
**1**	11	Yes	32
**2**	5	No	35
**5**	12	Yes	28
**7**	11	Yes	22
**8**	12	Yes	26
**10**	12	Yes	28
**11**	9	No	30
**12**	12	Yes	30
**13**	10	No	32
**14**	7	No	39
**16**	11	Yes	33
**17**	8	No	29
**18**	8	No	32

***Supplementary Table 6**. Comprehensive list of metabolites showing differential abundance in serum (FDR < 0.05) between inclusion body myositis patients (n=15) and healthy controls (n=5). Determined using limma ebayes functions with corrections for multiple comparisons.*

Compound	log2FC	t	P.Value	FDR
Carnosine	-5.9977	-17.4821	2.16E-16	4.54E-14
Indole-3-acrylic acid	-7.1153	-9.03628	1.04E-09	1.10E-07
Propionylcarnitine	-5.6643	-8.78901	1.85E-09	1.13E-07
Tryptophan	-5.7199	-8.72275	2.16E-09	1.13E-07
Cysteine	2.3819	7.255014	7.67E-08	3.22E-06
Erythro-sphingosine 1-phosphate	-4.1924	-7.14547	1.01E-07	3.54E-06
Ribose	3.6385	6.5077	5.23E-07	1.57E-05
3-Methyladipic acid	-2.9911	-5.99019	2.05E-06	5.37E-05
3-Methoxybenzaldehyde	2.3405	4.092871	0.000338	0.007894
Palmitoyl sphingomyelin	5.6308	4.032391	0.000398	0.007894
2,3-Dihydroxypropanoic acid	-1.3961	-4.01323	0.000418	0.007894
4-Hydroxybutyric acid (GHB)	1.2727	3.984905	0.000451	0.007894
Bilirubin	2.2348	3.862302	0.000624	0.010084
Suberic acid	-1.6171	-3.70583	0.000942	0.014135
3-Indoxyl sulphate	1.5418	3.62473	0.001165	0.016307
3-Hydroxybutyric acid	1.7879	3.506821	0.001582	0.020762
Pseudouridine	1.3243	3.444123	0.001859	0.022969
4-Methylphenol	1.4739	3.334197	0.002464	0.028743
Aspartic acid	-1.2951	-3.26095	0.002967	0.032797
Chenodeoxycholic Acid	1.8407	3.168465	0.003746	0.039332
Alpha-ketoglutaric acid	1.2429	3.072104	0.004764	0.046409
Azelaic acid	-1.8917	-3.05555	0.004963	0.046409
Xanthine	6.7453	3.033934	0.005236	0.046409
9-Methyluric acid	1.9534	3.02872	0.005304	0.046409
Ecgonine	2.1381	3.003731	0.005641	0.047384

Supplementary Table 7A *Gene set enrichment analysis using KEGG pathways identified a comprehensive list of the most differentially expressed pathways during the lead-in period (baseline to week 16). All pathways with an adjusted p-value < 0.05 are displayed. Analysis performed with clusterprofiler gseKEGG function with settings: minGSSize = 10,   maxGSSize = 500, pvalueCutoff = 0.05, pAdjustMethod = "BH", verbose = TRUE). OXPHOS = Oxidative phosphorylation, VPCI = Viral protein interaction with cytokine*

Gene Set Enrichment Analysis: Baseline vs. Week 16 (Lead-in period)									
ID	Description	setSize	Enrichment Score	NES	p value	p adjust	q value	rank	Cluster name
hsa00020	Citrate cycle (TCA cycle)	21	-0.7441	-2.100	0.000045	3.07E-03	2.68E-03	2589	TCA cycle
hsa00190	Oxidative phosphorylation	66	-0.5447	-1.969	0.000034	3.07E-03	2.68E-03	3327	*OXPHOS*
hsa05150	Staphylococcus aureus infection	33	0.7036	1.947	0.000046	3.07E-03	2.68E-03	1477	
hsa04061	Viral protein interaction with cytokine and cytokinpe receptor	74	0.5919	1.913	0.000043	3.07E-03	2.68E-03	2925	*VPCI*
hsa04060	Cytokine-cytokine receptor interaction	188	0.4781	1.736	0.000037	3.07E-03	2.68E-03	2949	*VPCI*
hsa04714	Thermogenesis	135	-0.4259	-1.721	0.000125	6.96E-03	6.08E-03	3479	*OXPHOS*
hsa04062	Chemokine signaling pathway	144	0.4852	1.702	0.000181	8.62E-03	7.52E-03	3104	*VPCI*
hsa04657	IL-17 signaling pathway	63	0.5498	1.720	0.000679	2.83E-02	2.47E-02	1625	IL-17 signaling pathway
hsa00785	Lipoic acid metabolism	11	-0.7760	-1.810	0.001080	3.61E-02	3.15E-02	2589	TCA cycle
hsa00280	Valine, leucine and isoleucine degradation	35	-0.5735	-1.799	0.001085	3.61E-02	3.15E-02	2664	
hsa00532	Glycosaminoglycan biosynthesis - chondroitin sulfate / dermatan sulfate	16	0.7598	1.825	0.001242	3.76E-02	3.28E-02	2365	
hsa05144	Malaria	32	0.6506	1.786	0.001729	4.61E-02	4.02E-02	2759	IL-17 signaling pathway
hsa04640	Hematopoietic cell lineage	55	0.5640	1.721	0.001799	4.61E-02	4.02E-02	2862	
hsa00250	Alanine, aspartate and glutamate metabolism	19	-0.6841	-1.875	0.002033	4.62E-02	4.03E-02	1427	
hsa04670	Leukocyte transendothelial migration	71	0.5208	1.668	0.002089	4.62E-02	4.03E-02	3104	
hsa04514	Cell adhesion molecules	97	0.4884	1.617	0.002219	4.62E-02	4.03E-02	2457	

Supplementary Table 7B *Gene set enrichment analysis using KEGG pathways identified a comprehensive list of the most differentially expressed pathways during pioglitazone treatment (week 16 to week 32). All pathways with an adjusted p-value < 0.05 are displayed.. Analysis performed with clusterprofiler gseKEGG function with settings: minGSSize = 10, maxGSSize = 500, pvalueCutoff = 0.05, pAdjustMethod = "BH", verbose = TRUE. OXPHOS = Oxidative phosphorylation, IBD = Inflammatory bowel disease, VPCI = Viral protein interaction with cytokine, PM = Propanoate metabolism, RLA = Regulation of lipolysis in adipocytes, CMC = Cytoskeleton in muscle cells, ArgBio = Arginine biosynthesis.*

Gene Set Enrichment Analysis: Week 16 vs. Week 32 (Pioglitazone treatment)									
ID	Description	setSize	Enrichment	NES	p value	p adjust	q value	rank	Cluster
hsa00190	Oxidative phosphorylation	66	0.7216	2.727	1.00E-10	1.28E-08	1.00E-08	1892	OXPHOS
hsa05415	Diabetic cardiomyopathy	108	0.5761	2.416	1.15E-10	1.28E-08	1.00E-08	2070	OXPHOS
hsa04714	Thermogenesis	135	0.5426	2.338	1.00E-10	1.28E-08	1.00E-08	2781	OXPHOS
hsa03010	Ribosome	83	0.6011	2.391	1.26E-09	9.51E-08	7.43E-08	3941	
hsa04932	Non-alcoholic fatty liver disease	100	0.5746	2.367	1.43E-09	9.51E-08	7.43E-08	1953	OXPHOS
hsa05016	Huntington disease	173	0.4666	2.093	2.08E-09	1.15E-07	9.00E-08	1892	OXPHOS
hsa05208	Chemical carcinogenesis - reactive oxygen species	130	0.5239	2.246	4.60E-09	2.19E-07	1.71E-07	2122	OXPHOS
hsa05022	Pathways of neurodegeneration - multiple diseases	297	0.3913	1.880	8.99E-09	3.74E-07	2.92E-07	1803	OXPHOS
hsa05010	Alzheimer disease	235	0.4127	1.939	4.01E-08	1.48E-06	1.16E-06	1892	OXPHOS
hsa00020	Citrate cycle (TCA cycle)	21	0.8128	2.410	5.22E-08	1.58E-06	1.23E-06	1925	TCA Cycle
hsa05014	Amyotrophic lateral sclerosis	221	0.4183	1.944	5.20E-08	1.58E-06	1.23E-06	1755	OXPHOS
hsa01200	Carbon metabolism	67	0.5983	2.260	1.68E-07	4.65E-06	3.63E-06	2086	TCA Cycle
hsa05012	Parkinson disease	148	0.4524	1.974	3.10E-07	7.93E-06	6.19E-06	1798	OXPHOS
hsa04910	Insulin signaling pathway	97	0.5119	2.078	5.14E-07	1.22E-05	9.55E-06	2913	AMPK
hsa04974	Protein digestion and absorption	71	-0.6236	-2.098	7.32E-07	1.63E-05	1.27E-05	2199	
hsa04061	*VPCI* and cytokine receptor	74	-0.6022	-2.038	1.68E-06	3.50E-05	2.74E-05	3309	*VPCI*
hsa03320	PPAR signaling pathway	46	0.6096	2.114	4.05E-06	7.94E-05	6.20E-05	3155	
hsa00280	Valine, leucine and isoleucine degradation	35	0.6571	2.195	1.21E-05	0.000212	0.000166	2909	*PM*
hsa04060	Cytokine-cytokine receptor interaction	188	-0.4695	-1.787	1.21E-05	0.000212	0.000166	3309	*VPCI*
hsa04923	Regulation of lipolysis in adipocytes	35	0.6541	2.185	1.38E-05	0.000229	0.000179	1953	*RLA*
hsa00650	Butanoate metabolism	18	0.7751	2.229	1.96E-05	0.00031	0.000242	1330	
hsa04062	Chemokine signaling pathway	144	-0.4900	-1.808	2.52E-05	0.000382	0.000298	2341	*VPCI*
hsa00620	Pyruvate metabolism	26	0.7016	2.208	3.02E-05	0.000437	0.000341	3175	TCA Cycle
hsa04723	Retrograde endocannabinoid signaling	88	0.4670	1.881	3.99E-05	0.000554	0.000432	1900	OXPHOS
hsa05020	Prion disease	146	0.4028	1.756	4.16E-05	0.000554	0.000432	1784	OXPHOS
hsa00640	Propanoate metabolism	18	0.7492	2.155	9.68E-05	0.001194	0.000933	3175	*PM*
hsa00250	Alanine, aspartate and glutamate metabolism	19	0.7241	2.102	9.58E-05	0.001194	0.000933	1508	*ArgBio*
hsa04512	ECM-receptor interaction	57	-0.5780	-1.886	0.000112	0.001326	0.001036	1234	*CMC*
hsa01210	2-Oxocarboxylic acid metabolism	17	0.7329	2.085	0.000161	0.001845	0.001441	2003	TCA Cycle
hsa04920	Adipocytokine signaling pathway	51	0.5129	1.853	0.000228	0.002536	0.00198	2991	AMPK
hsa00785	Lipoic acid metabolism	11	0.8115	1.965	0.000262	0.002722	0.002125	2003	TCA Cycle
hsa04152	AMPK signaling pathway	85	0.4486	1.801	0.000258	0.002722	0.002125	2072	AMPK
hsa04514	Cell adhesion molecules	97	-0.5044	-1.777	0.000313	0.003155	0.002463	3247	
hsa04964	Proximal tubule bicarbonate reclamation	18	0.7108	2.044	0.000499	0.004884	0.003814	1074	
hsa04922	Glucagon signaling pathway	66	0.4739	1.791	0.000938	0.008926	0.006969	1741	AMPK
hsa04931	Insulin resistance	73	0.4224	1.630	0.001866	0.017263	0.013478	1981	AMPK
hsa05140	Leishmaniasis	39	-0.5616	-1.690	0.002507	0.022077	0.017237	3059	IBD
hsa04150	mTOR signaling pathway	108	0.3677	1.542	0.002519	0.022077	0.017237	2484	
hsa00630	Glyoxylate and dicarboxylate metabolism	18	0.6576	1.891	0.002691	0.022979	0.017942	2272	
hsa05130	Pathogenic Escherichia coli infection	128	-0.4265	-1.547	0.003011	0.025068	0.019573	4528	
hsa04146	Peroxisome	52	0.4562	1.645	0.003393	0.027556	0.021515	3012	
hsa05321	Inflammatory bowel disease	36	-0.5639	-1.661	0.004609	0.034709	0.0271	3059	IBD
hsa04623	Cytosolic DNA-sensing pathway	39	-0.5419	-1.631	0.004602	0.034709	0.0271	2415	
hsa04610	Complement and coagulation cascades	42	-0.5348	-1.627	0.00469	0.034709	0.0271	2511	
hsa04022	cGMP-PKG signaling pathway	109	0.3420	1.433	0.004683	0.034709	0.0271	1967	*RLA*
hsa00532	Glycosaminoglycan biosynthesis chondroitin sulfate / dermatan sulfate	16	-0.7122	-1.774	0.005037	0.035143	0.027439	2400	
hsa00010	Glycolysis / Gluconeogenesis	34	0.5276	1.755	0.005043	0.035143	0.027439	2149	
hsa04211	Longevity regulating pathway	64	0.4147	1.559	0.005066	0.035143	0.027439	2781	AMPK
hsa00220	Arginine biosynthesis	12	0.7198	1.795	0.00557	0.03785	0.029553	901	ArgBio
hsa05146	Amoebiasis	72	-0.4686	-1.581	0.006278	0.041814	0.032648	3291	IBD
hsa05323	Rheumatoid arthritis	56	-0.4922	-1.596	0.006498	0.042113	0.032881	3059	IBD
hsa04820	Cytoskeleton in muscle cells	141	-0.4053	-1.489	0.006576	0.042113	0.032881	1327	*CMC*
hsa00565	Ether lipid metabolism	29	-0.5712	-1.612	0.007202	0.04441	0.034675	2779	

hsa04137	Mitophagy - animal	75	0.3980	1.553	0.007178	0.04441	0.034675	4856	
hsa00982	Drug metabolism - cytochrome P450	23	0.5747	1.741	0.007417	0.044907	0.035062	1317	
hsa04110	Cell cycle	130	-0.4106	-1.497	0.007878	0.046843	0.036574	1967	

Supplementary Table 8A. *Integrated pathway analysis of transcriptomic and metabolomic data during the lead-in period (baseline to week 16) .All pathways with an adjusted p-value < 0.05 are displayed. Determined using multiGSEA package and function.* *VPCI = Viral protein interaction with*

Integrated Pathway Analysis of Transcriptomic and Metabolomic data: Baseline vs. Week 16 (Lead-in period)							
	KEGG pathway	transcriptome_pval	transcriptome_padj	metabolome_pval	metabolome_padj	combined_pval	combined_padj
1	Herpes simplex virus 1 infection	2.25462094517349e-06	0.000728	NA	NA	2.25462094517349e-06	0.000733
2	Cytokine-cytokine receptor interaction	1.01300603180482e-05	0.001091	NA	NA	1.01300603180482e-05	0.001646
3	Pathogenic Escherichia coli infection	2.72183172748095e-05	0.002198	NA	NA	2.72183172748095e-05	0.002949
4	Influenza A	5.53467571015798e-05	0.003575	NA	NA	5.53467571015798e-05	0.004497
5	Rap1 signaling pathway	8.81786797716557e-05	0.004747	NA	NA	8.81786797716557e-05	0.005732
6	Leukocyte transendothelial migration	0.000131	0.00582	NA	NA	0.000131	0.007097
7	Chemokine signaling pathway	0.000176	0.00582	NA	NA	0.000176	0.00732
8	Hepatitis C	0.00018	0.00582	NA	NA	0.00018	0.00732
9	Human papillomavirus infection	0.000211	0.006185	NA	NA	0.000211	0.007606
10	*VPCI* and cytokine receptor	0.000286	0.007702	NA	NA	0.000286	0.0093
11	Sphingolipid signaling pathway	0.005488	0.052138	0.015578	0.175995	0.000446	0.013186
12	PI3K-Akt signaling pathway	0.000487	0.011328	NA	NA	0.000487	0.013194
13	Valine, leucine and isoleucine degradation	0.000173	0.00582	0.194274	0.791667	0.000846	0.018281
14	ECM-receptor interaction	0.000805	0.016254	NA	NA	0.000805	0.018281
15	Cytosolic DNA-sensing pathway	0.0009	0.017099	NA	NA	0.0009	0.018281
16	Measles	0.000792	0.016254	NA	NA	0.000792	0.018281
17	Proteoglycans in cancer	0.000981	0.0176	NA	NA	0.000981	0.01875
18	JAK-STAT signaling pathway	0.001102	0.018729	NA	NA	0.001102	0.019892
19	Neutrophil extracellular trap formation	0.001168	0.018867	NA	NA	0.001168	0.019983
20	Focal adhesion	0.001322	0.019411	NA	NA	0.001322	0.021485
21	MicroRNAs in cancer	0.001753	0.024623	NA	NA	0.001753	0.027135
22	Efferocytosis	0.004411	0.04343	0.096907	0.511455	0.002797	0.037883
23	Regulation of actin cytoskeleton	0.002598	0.034963	NA	NA	0.002598	0.037883
24	Yersinia infection	0.002751	0.035338	NA	NA	0.002751	0.037883
25	Glycosaminoglycan biosynthesis - chondroitin sulfate / dermatan sulfate	0.002965	0.035466	NA	NA	0.002965	0.038469
26	Gap junction	0.003098	0.035739	NA	NA	0.003098	0.038469
27	Natural killer cell mediated cytotoxicity	0.003348	0.036958	NA	NA	0.003348	0.038469
28	Inflammatory mediator regulation of TRP channels	0.014654	0.091025	0.049817	0.378694	0.003412	0.038469
29	AGE-RAGE signaling pathway in diabetic complications	0.003433	0.036958	NA	NA	0.003433	0.038469
30	Galactose metabolism	0.01404	0.088919	0.055215	0.378694	0.003663	0.039681
31	Human T-cell leukemia virus 1 infection	0.003916	0.040797	NA	NA	0.003916	0.04105

Supplementary Table 8B. *Integrated pathway analysis of transcriptomic and metabolomic data during the lead-in period during the treatment period with piogiltazone (week 16 to week 32). All pathways with an adjusted p-value < 0.05 are displayed. Determined using multiGSEA package and function. VPCI = Viral protein interaction with cytokine, GAG=* Glycosaminoglycan

Integrated Pathway Analysis of Transcriptomic and Metabolomic data: week 16 to week 32 (Pioglitazone treatment)							
	KEGG pathway	transcriptome_pval	transcriptome_padj	metabolome_pval	metabolome_padj	combined_pval	combined_padj
1	ECM-receptor interaction	1.15533683140672e-06	0.000124	NA	NA	1.15533683140672e-06	0.000188
2	Cell adhesion molecules	4.08805282606434e-05	0.002201	NA	NA	4.08805282606434e-05	0.004429
3	Valine, leucine and isoleucine degradation	6.6922298718883e-07	0.000108	0.367113	0.788827	0.000127	0.00564
4	PPAR signaling pathway	0.0001	0.002697	NA	NA	0.0001	0.00564
5	Cytokine-cytokine receptor interaction	0.000156	0.003604	NA	NA	0.000156	0.00564
6	*VPCI* and cytokine receptor	9.58609355234908e-05	0.002697	NA	NA	9.58609355234908e-05	0.00564
7	Alzheimer disease	0.000131	0.003256	NA	NA	0.000131	0.00564
8	Pathways of neurodegeneration	1.89892053245008e-05	0.001533	0.161157	0.733591	0.000151	0.00564
9	Rap1 signaling pathway	0.000244	0.005259	NA	NA	0.000244	0.007937
10	Propanoate metabolism	3.57377967719602e-05	0.002201	0.188017	0.733591	0.000297	0.008783
11	*GAG* biosynthesis - chondroitin sulfate / dermatan sulfate	0.000572	0.010394	NA	NA	0.000572	0.014279
12	Chemokine signaling pathway	0.000579	0.010394	NA	NA	0.000579	0.014279
13	AMPK signaling pathway	0.000659	0.011204	NA	NA	0.000659	0.014279
14	Parkinson disease	6.30437866917799e-05	0.002697	0.23166	0.733591	0.00062	0.014279
15	Influenza A	0.000743	0.011701	NA	NA	0.000743	0.015083
16	HPV infection	0.000797	0.011701	NA	NA	0.000797	0.015237
17	Calcium signaling pathway	0.001073	0.014863	NA	NA	0.001073	0.019377
18	Mitophagy - animal	0.001589	0.017638	NA	NA	0.001589	0.024201
19	PI3K-Akt signaling pathway	0.001578	0.017638	NA	NA	0.001578	0.024201
20	Focal adhesion	0.001475	0.017638	NA	NA	0.001475	0.024201
21	Complement and coagulation cascades	0.001638	0.017638	NA	NA	0.001638	0.024201
22	Pathogenic Escherichia coli infection	0.001826	0.01903	NA	NA	0.001826	0.025807
23	Citrate cycle (TCA cycle)	8.85166188655296e-05	0.002697	0.367769	0.788827	0.001924	0.02606
24	Carbon metabolism	8.14421960670092e-05	0.002697	0.382932	0.788827	0.002009	0.026116
25	Efferocytosis	0.008433	0.055741	0.059846	0.733591	0.002635	0.03294
26	Alanine, aspartate and glutamate metabolism	0.006583	0.046224	0.077778	0.733591	0.002914	0.034786
27	Pyruvate metabolism	7.48740857995314e-05	0.002697	0.462185	0.798319	0.002997	0.034786
28	Retrograde endocannabinoid signaling	0.001578	0.017638	0.18595	0.733591	0.003275	0.035728
29	Insulin signaling pathway	0.003298	0.031311	NA	NA	0.003298	0.035728
30	Arginine and proline metabolism	0.08589	0.261282	0.007083	0.441967	0.003458	0.035781
31	Cytosolic DNA-sensing pathway	0.003523	0.031311	NA	NA	0.003523	0.035781
32	Autophagy - animal	0.003684	0.031311	NA	NA	0.003684	0.036278
33	*GAG* degradation	0.0039	0.032301	NA	NA	0.0039	0.037281
34	Fatty acid degradation	0.004368	0.035275	NA	NA	0.004368	0.040563
35	Gap junction	0.004648	0.036621	NA	NA	0.004648	0.041965
36	Axon guidance	0.005073	0.039015	NA	NA	0.005073	0.044562
37	Butanoate metabolism	0.002292	0.022434	0.224532	0.733591	0.005545	0.04621
38	Lipoic acid metabolism	0.000513	0.010358	0.375783	0.788827	0.005457	0.04621
39	Fructose and mannose metabolism	0.111111	0.293795	0.009305	0.441967	0.00575	0.046717

Summary of Changes to Protocol:

- Version 1.0, approved 24-FEB-2018

- Version 2.0, approved 17-MAY-2018 (Protocol Amendment 1)
    - Changes from Version 1.0:
        - Secondary endpoints of the Fi-2, m-TUG, and 6-minute walk test added

- Version 3.0, approved 10-JUN-2018 (Protocol Amendment 2)
    - Changes from Version 2.0:
        - Specified the heart failure exclusion criteria to be the New York Heart Association class II-IV

- Version 4.0, approved 27-AUG-2019 (Protocol Amendment 3)
    - Changes from Version 3.0:
        - Expanded the recruitment goal to 20 participants

- Version 5.0, approved 9-FEB-2022 (Protocol Amendment 4)
    - Changes from Version 4.0:
        - Added a secondary endpoint looking at changes in metabolites with pioglitazone
        - Included control muscle biopsies and serum samples to be used as a comparison with IBM specimens.

- Version 6.0, approved 23-APRIL-2025 (Protocol Amendment 5)
    - Changes from Version 5.0:
        - Added data deposition in The Vivli repository.

JHM IRB - eForm A – Protocol

An Open-Label Pilot Study of Pioglitazone in Sporadic Inclusion Body Myositis

****

1. Abstract

Sporadic inclusion body myositis (sIBM) is an idiopathic myopathy that presents in older age with progressive muscle weakness and atrophy and often leads to severe functional impairment and disability. The etiology is poorly understood and there is no known pharmacologic treatment. Multiple studies have demonstrated prominent mitochondrial impairment in skeletal muscle from sIBM patients. Multiple mitochondrial genes involved in oxidative phosphorylation are down-regulated as well as PGC-1α, a master regulator of mitochondrial biogenesis. Pioglitazone is an FDA-approved drug for type II diabetes that is known to increase PGC-1 α in muscle from patients with diabetes.

The purpose of this research project is to test whether mitochondrial function and the expression of genes in the oxidative phosphorylation and mitochondrial pathways in skeletal muscle from sIBM patients are altered in response to pioglitazone. We hypothesize that pioglitazone will upregulate PGC-1 α in skeletal muscle of sIBM patients and ameliorate some of the mitochondrial deficits. Findings from this project will add novel insights into the molecular mechanisms that mediate mitochondrial dysfunction in sIBM and shed light on a potential therapeutic option.

2. Objectives and Endpoints

Primary Objective: To determine the effect of pioglitazone on mitochondrial function and the expression of PGC-1α and other genes in the oxidative phosphorylation and mitochondrial pathways in skeletal muscle of patients with sIBM.

Secondary Objectives:

1. To evaluate the effects of pioglitazone on muscle strength and endurance in patients with sIBM.
2. To evaluate the effects of pioglitazone on disability and patient-reported outcomes in patients with sIBM.
3. To assess the architecture of skeletal muscle in patients with sIBM pre- and post- pioglitazone treatment, and correlate ultrasound findings with clinical outcomes.
4. To investigate the effect of pioglitazone on the incidence of self-reported falls.
5. To study the effects of pioglitazone on serum biomarkers including creatine kinase (CK)
6. To assess the safety and tolerability of pioglitazone in patients with sIBM.
7. To study the relationship between clinical changes in patients with sIBM and changes in mitochondrial function and expression of mitochondrial biomarkers.
8. To compare mitochondrial function and mitochondrial gene expression in affected and unaffected muscle in sIBM.
9. To assess changes in metabolomics before and after pioglitazone treatment

Primary Endpoint: Change in expression of PGC-1α and other genes in the oxidative phosphorylation and mitochondrial function pathways. This will be assessed by the change in mitochondrial mRNA and/or protein expression after four months of treatment with pioglitazone, as compared to the change in these biomarkers during the preceding 4 month lead-in period.

Secondary Endpoints:

1. Change in strength as measured by manual muscle testing, quantitative dynamometry, the functional index-2 (Fi-2), the modified timed up-and-go test (m-TUG), and the 6-minute walk test
2. Change in the IBM-Functional Rating Scale (IBM-FRS)
3. Change in architecture of skeletal muscle (e.g. muscle size and echointensity) assessed by ultrasound
4. The number of falls that patient self-reports, occurring during the 4 month lead-in period compared to the period of pioglitazone treatment
5. Change in serum creatine kinase
6. The occurrence of treatment-related adverse events or intolerance to therapy
7. Expression of PGC-1α and other genes in the oxidative phosphorylation and mitochondrial function pathways, as assessed by change in mRNA expression, and clinical outcomes including strength and patient reported outcomes
8. Difference in mitochondrial function (respiration and glycolysis) and expression of genes involved in oxidative phosphorylation and mitochondrial function in affected and unaffected muscle.
9. Difference in metabolomic markers before and after drug treatment

3. Background

Sporadic inclusion body myositis (sIBM)

Sporadic inclusion body myositis (sIBM) is one of the idiopathic inflammatory myopathies. It is a rare disease with an estimated prevalence of 5-9 cases per million adults [1]. Unlike the other inflammatory myopathies polymyositis and dermatomysitis, sIBM almost universally affects older people above the age of 50 and is refractory to immunosuppressive therapies including prednisone. sIBM causes progressive muscle atrophy and weakness, resulting in frequent falls and significant disability [2]. There is no known pharmacologic treatment for sIBM.

The underlying pathophysiology of sIBM remains unclear. It is currently controversial whether sIBM is primarily an inflammatory or degenerative myopathy. The presence of specific autoantibodies and cytotoxic T cells surrounding myofibers has led to the hypothesis that it is an inflammatory myopathy. However, unlike other autoimmune conditions, sIBM afflicts an older population and patients generally do not respond to immunosuppression. Abnormal cellular protein accumulation has also been identified in sIBM and suggests that sIBM may share certain common pathologic mechanisms with degenerative diseases such as frontotemporal dementia.

Mitochondrial Deficits in sIBM

There is accumulating evidence that patients with sIBM have profound deficits in mitochondrial function in skeletal muscle. Skeletal muscle from patients with sIBM have increased accumulation of ragged red fibers, a marker of impaired mitochondrial oxidative metabolism, which is notably absent in muscle biopsies from patients with polymyositis or dermatomyositis [3]. Muscle from sIBM patients also has a deficiency in activity of the electron transport protein cytochrome C oxidase [3] and frequently demonstrate large-scale mitochondrial DNA (mtDNA) deletions [4]. Unpublished work from our group has demonstrated that expression of multiple genes involved in

mitochondrial oxidative phosphorylation and mitochondrial function are markedly downregulated in skeletal muscle of sIBM patients. These observations support the theory that mitochondrial dysfunction in skeletal muscle is involved in the pathogenesis of sIBM.

The mitochondrial dysfunction theory in sIBM is further supported by the consistent clinical observation that endurance exercise may improve the clinical outcome of sIBM [5–8]. Exercise is recognized to improve mitochondrial biogenesis and function [9], which raises the intriguing possibility that exercise may improve outcomes in sIBM by enhancing mitochondrial function. Therefore, mitochondrial dysregulation and the upstream molecular pathways that impact mitochondrial function and biogenesis are promising therapeutic targets for sIBM.

Rationale

The molecular pathways that are upregulated by exercise and their downstream effects on mitochondria are well understood. The transcriptional co-activator peroxisome proliferator-activated receptor γ co- activator 1α (PGC-1α) is a master regulator that plays a key role in cellular energy homeostasis and metabolism. Endurance exercise activates AMPK, which in turn activates PGC-1α in human skeletal muscle and leads to down-stream mitochondrial biogenesis [10]. We have found that PGC-1α mRNA and protein expression are disproportionately down-regulated in skeletal muscle of sIBM patients. These results are consistent with findings from another group that has also demonstrated decreased expression of PGC1α, AMP-activated protein kinase (AMPK), and other proteins involved in mitochondrial function or biogenesis in skeletal muscle from patients with sIBM [11]. Therefore, pharmacologic modulation of this pathway may enhance mitochondrial biogenesis and therefore improve muscle strength in sIBM.

Pioglitazone is a generic and well-tolerated drug of the thiazolidinedione (TZD) class that is an insulin sensitizing agent approved for the treatment of type 2 diabetes. The pharmacologic target of pioglitazone is the nuclear receptor peroxisome proliferator-activated receptor gamma (PPARγ), which modulates the expression of multiple genes involved in insulin signaling and glucose metabolism in peripheral tissues. Pioglitazone increases expression of activated AMPK and PGC-1α in human skeletal muscle of patients with diabetes [12], resulting in increased mitochondrial biogenesis and improved exercise capacity and mitochondrial function [13,14]. Pioglitazone's effects on the expression of genes that regulate mitochondrial function in skeletal muscle is thought to be one of the primary mechanisms through which it lowers blood glucose and ameliorates insulin resistance in diabetes.

The purpose of this study is to determine if pioglitazone activates the AMPK and PGC-1α pathway in skeletal muscle of sIBM patients and ameliorates the mitochondrial deficits and dysfunction. We also seek to assess the safety and feasibility of pioglitazone treatment in patients with sIBM. If this study is successful, this study may lead to the development of the first effective therapy for patients with sIBM.

Correlative Studies Background

Pioglitazone has been widely studied in adipose and muscle tissue and is recognized to have numerous effects on pathways involved in mitochondrial biogenesis and function. In human adipose tissue from patients with diabetes, pioglitazone increases mitochondrial copy number, mRNA expression of cytochrome C, and citrate synthase activity [20], which is a mitochondrial

protein that in muscle has been shown to be a marker of mitochondrial aerobic capacity [21]. Pioglitazone increases mRNA expression of PGC-1alpha and its downstream target mitochondrial transcription factor A (mtTFA) in adipose tissue, both of which are key regulators of mitochondrial biogenesis and oxidative metabolism [14].

In skeletal muscle from patients with polycystic ovarian syndrome (PCOS), pioglitazone has similar effects as in adipose tissue and significantly upregulates genes involved in mitochondrial oxidative phosphorylation (OXPHOS). In muscle from diabetic patients, pioglitazone increases expression of PGC-1alpha and increases phosphorylation of AMP-activated protein kinase (AMPK) [12]. AMPK is a key regulator of cellular energy homeostasis that is activated during exercise and has down-stream effects on mitochondrial content [22]. Nuclear Respiratory Factor-1 (NRF-1), another transcription factor involved in mitochondrial biogenesis that is down-stream of AMPK [23], is upregulated by pioglitazone in cultured myotubes [24] and in muscle from Zuker diabetic rats [25], although this effect has not been observed in human clinical trials of pioglitazone in patients with diabetes [12,20]. In skeletal muscle from patients with diabetes, pioglitazone upregulates a number of nuclear-encoded mitochondrial genes that encode for scavenging enzymes that help to protect mitochondria against reactive oxygen species, including cytochrome c oxidase subunit V1c (COX6C), NADH dehydrogenase 1-alpha subcomplex, 5 (NDUFA5), cAMP-responsive element binding protein 1 (CREB1), myocyte enhancer factor 2C (MEF2C), and sirtuin 1 (SIRT1) [12]. Functionally, pioglitazone improves exercise capacity in diabetic mice, which is mediated by improved mitochondrial function and decreased oxidative stress [13].

The ability of pioglitazone to ameliorate mitochondrial deficits in sIBM skeletal muscle is unknown. In this study, we will perform correlative studies on muscle tissue obtained from patients with sIBM before and after treatment with pioglitazone. We will use a Seahorse machine to measure mitochondrial respiration and glycolysis as a measure of mitochondrial function. We will also measure mitochondrial DNA (mtDNA). In collaboration with Dr. Andrew Mammen at the NIH, we will perform RNA sequencing and use Ingenuity Pathway Analysis to assess for broad changes in genes involved in mitochondrial oxidative phosphorylation (OXPHOS) and mitochondrial function after treatment with pioglitazone. In particular, we will use RNA sequencing to measure changes in expression of genes coding for important mitochondrial regulators, including PGC-1 alpha, cytochrome C, mtTFA, cytochrome c oxidase subunit V1c, NADH dehydrogenase 1-alpha subcomplex, 5, cAMP-responsive element binding protein 1, myocyte enhancer factor 2C, sirtuin 1, NRF-1, NRF-2, mitochondrial transcription factors B1 and B2, estrogen receptor-alpha and total AMPK. We will also measure changes in gene expression of the NR4A family, PDK4, ANGPTL4, and SLC22A5 genes, all of which are related to PPAR signaling and are induced by exercise [26]. If there is sufficient tissue, western blot analysis will be used to measure changes in protein expression of phosphorylated AMPK before and after treatment. **Please see page 7 for a full description of our NIH/NIAMS collaboration with Dr. Andrew Mammen, MD, PhD, Associate Investigator.**

We will use ultrasound to assess for any changes in skeletal muscle architecture and we will correlate these changes in architecture with functional improvement or decline. There are several published reports [27] [28] about the utility of ultrasound in highlighting changes in echogenicity of the gastrocnemius muscle [29] and the flexor digitorum profundus relative to flexor carpi ulnaris [27] in patients with sIBM. In our experience, muscle affected by sIBM has characteristic changes in size

and muscle quality (echointensity) compared to other inflammatory myopathies. For this study, we will be imaging the muscle groups that are either most affected in sIBM or show the most distinct changes. This includes but is not limited to the flexor digitorum profundus (FDP), rectus femoris, vastus lateralis, gastrocnemius, and tibialis anterior.

4. Study Procedures

This is a 52-week, Phase 1, open-label, single center, proof of concept study of FDA-approved pioglitazone in adult patients with sIBM. The trial is designed to evaluate mitochondrial function and the expression of genes in the oxidative phosphorylation and mitochondrial pathways in skeletal muscle as well as the safety, tolerability, and treatment effect of pioglitazone in these patients. The trial consists of a:

- 4-week screening period;
- 16-week "lead-in" period during which all subjects are observed off-treatment. At Week 16, all subjects will be started on pioglitazone at a dose of 30 mg daily. The dose will be uptitrated to a goal dose of 45 mg daily after 2 weeks;
- 32-week treatment period with all subjects on 45 mg daily dose of pioglitazone.

<u>Please see Appendix I: Schedule of Assessments.</u>
<u>For scheduling purposes, study procedures for Visit 2/Week 0, Visit 3/Week 16, Visit 5/Week 32 may be conducted over 2 consecutive days. The study team would like to maximize scheduling flexibility on days that require different investigators for functional assessments and muscle biopsies.</u>

<u>Recruitment:</u>
Participants will be recruited during routine clinic visits at the Johns Hopkins Myositis Center. Participants may also be recruited from other patient populations at the Johns Hopkins Hospital or the myositis community through the use of advertisements or by engaging previous study participants who gave authorization to be contacted regarding future studies. Study procedures will be conducted at the Johns Hopkins Bayview Medical campus. We anticipate enrolling up to 20 adult patients with sIBM. Up to 30 patients may be consented in order to complete enrollment. Interested study participants will be evaluated to ensure they meet basic criteria for participation (see Section 3 for patient selection).

<u>Visit 1: Screening and Informed Consent</u>
During the first scheduled research visit, the protocol will be discussed in detail with the subject and written documentation will be obtained of the subject's consent to participate in the study and subject's agreement to be contacted about future research studies. We will also include consent form language so that remaining tissue material collected throughout the study may be de-identified and stored for use in future studies. The participant will be given sufficient time to read the consent form and ask any questions before signing the consent. Once informed consent is obtained, subjects will be screened for eligibility for entry into the study based on the inclusion and exclusion criteria. Screening procedures will be performed within 4 weeks prior to Baseline (Visit 2). The following evaluations will be done:

- Medical history and demographics review
- Physical examination with vital signs, height, weight

- Assessment of the patient's ability to rise from a chair unassisted and to ambulate more than 20 feet without support from another person, wall or furniture.
- Clinical laboratory tests (see Appendix I: Schedule of Assessments). Routine clinical laboratory samples will be tested at a Johns Hopkins CLIA-certified laboratory.
- Medication review. If the participant is on aspirin, other blood thinners, or anti-inflammatories (NSAIDS) they will be asked to refrain from taking these medications for 5 days prior to the baseline muscle biopsy.
- Muscle ultrasound will be done to confirm if subject has muscle suitable for biopsy.

The participant's next study visit will be scheduled as soon as eligibility is confirmed.

Visit 2: Baseline
Commencing at baseline, subjects will be observed off treatment for 16 weeks. This period off treatment will serve as the control for each patient, particularly with regard to changes in mitochondrial function and RNA expression of mitochondrial biomarkers in muscle tissue.

- Physical examination with vital signs
- Medication review
- Adverse events review
- Participants will be given a falls diary to record any falls
- Clinical assessments: Investigators will conduct clinical assessments at study visits to rate severity of muscle involvement. Disease-specific outcome measures will include manual muscle testing using the Medical Research Council scale (MRCS), quantitative muscle testing using dynamometry, hand-grip strength, functional measures, the IBM-Functional Rating Scale (IBM-FRS), and muscle-associated enzymes (CK).
- Muscle ultrasound: We will scan several muscle groups of the upper and lower extremities with the use of ultrasound to assess for changes in muscle size and muscle quality (echointensity). Quantitative measures of muscle depth, cross sectional area and echointensity will be obtained throughout the study.
- Needle muscle biopsy of affected muscle: At the baseline visit, subjects will undergo a needle muscle biopsy of affected muscle from either the upper or lower extremity (e.g. the vastus lateralis or tibialis anterior) in an outpatient clinic setting. Injectable anesthetics will be used for the procedure. After removal of small amounts of tissue (milligrams) for molecular studies, subjects may experience minimal bleeding, mild bruising, pain and discomfort.
- Optional needle muscle biopsy of unaffected or less affected muscle: To compare mitochondrial function and mitochondrial gene expression in affected and unaffected muscle, subjects will be asked to undergo an additional biopsy of unaffected muscle. For the purposes of this study, the biopsy of unaffected muscle may be completed at Visit 2 or Visit 3. Investigators are particularly interested in examining classically severely affected muscles in IBM (like the quadriceps) and seeing if there are any differences between muscles that are generally spared until late-stage disease (like the hamstrings).
- 50 cc of blood will be collected for laboratory study. Serum will be isolated and samples coded, de-identified, and stored.

Laboratory studies:

The Johns Hopkins Division of Rheumatology and Department of Neurology/Neuromuscular Division will conduct laboratory studies on the tissue specimens. In addition to the investigation of metabolomics and mitochondrial function before and after pioglitazone treatment, studies may also include ELISAs, protein arrays, flow cytometry, real-time quantitative PCR, among others to detect potential exploratory biomarkers in muscle, blood, and sera. As controls, we will utilize 10 muscle biopsy specimens that are histologically normal from the Myositis Research Registry database (NA_00007454) and 10 serum samples from healthy controls (IRB00066509). **No cell lines will be created as part of this study.** Participants will be assigned a unique identifier number. All data and samples will be coded using the unique identifier number during the study and will not include any personal identifiers. Only Johns Hopkins investigators will maintain and have access to the re-identification key. Any remaining tissue material may be de-identified and stored for use in future studies.

Collaboration with Andrew Mammen, MD, PhD, Associate Investigator:
As part of the study, we aim to use RNA sequencing and Ingenuity Pathway Analysis to assess for broad changes in genes involved in mitochondrial oxidative phosphorylation (OXPHOS) and mitochondrial function after treatment with pioglitazone. In particular, we will use RNA sequencing to measure changes in expression of genes coding for important mitochondrial regulators, including PGC-1 alpha, cytochrome C, mtTFA, cytochrome c oxidase subunit V1c, NADH dehydrogenase 1-alpha subcomplex, 5, cAMP-responsive element binding protein 1, myocyte enhancer factor 2C, sirtuin 1, NRF-1, NRF-2, mitochondrial transcription factors B1 and B2, estrogen receptor-alpha and total AMPK. We will also measure changes in gene expression of the NR4A family, PDK4, ANGPTL4, and SLC22A5 genes, all of which are related to PPAR signaling and are induced by exercise [26]. Accordingly, we seek to collaborate with Dr. Mammen, an expert in muscle pathology with extensive experience with RNAseq data analysis. Additionally, we have successfully collaborated already in generating the preliminary data shown above.

Muscle tissue will be sent to the laboratory of Andrew Mammen, M.D., Ph.D. at the Muscle Disease Unit at the NIH/NIAMS in Bethesda, Maryland. Participants will be assigned a unique identifier number. All muscle samples will be coded by the JHU study team using the unique identifier number, so Dr. Mammen will only receive coded samples with no personal identifiers. Muscle tissue samples sent from JHU to the off-site NIH will be shipped and transported in accordance with biosafety shipping policies for processing and laboratory analysis by Dr. Mammen. Dr. Mammen will be responsible for RNA isolation, sequencing on the samples, as well as analysis. Raw RNAseq data and aggregated results will be shared with JHU. Each site will have access to coded clinical data (for example, responders vs. non-responders to treatment). Both institutions will collaborate in data analysis and manuscript preparation. **Because this is a FDA-regulated investigational drug trial and not considered "exempt human subjects research," Dr. Mammen will also seek IRB review and approval of this protocol at his site in order to collaborate in the research under the protocol.**

Visit 3: Week 16
Following the 16-week "lead-in" observation period, the Investigational Drug Pharmacy at Bayview will dispense study drug for a four-month period. The starting dose of pioglitazone will be 30 mg orally daily. Study drug may not be administered until all of the following procedures have been completed:

- Physical examination with vital signs
- Medication review

- Adverse events review
- Clinical laboratory tests (see Appendix I: Schedule of Assessments)
- Participants will be given a new falls diary
- Clinical assessments
- Muscle ultrasound
- Needle muscle biopsy of affected muscle. If the participant is on aspirin, other blood thinners, or anti-inflammatories (NSAIDS) they will be asked to refrain from taking these medications for 5 days prior to the Week 16 muscle biopsy.
- The optional needle biopsy of unaffected muscle (described above at Visit 2) may be conducted at Visit 3 if not already done.
- 50 cc of blood will be collected and serum will be isolated and stored for laboratory study
- Subjects will receive written and verbal instructions for drug administration and will be asked to maintain a medication diary to monitor treatment compliance and return all used medication bottles to the study team for disposal.

Phone visit: Week 18

Participants will be contacted by phone 2 weeks after starting study drug to inquire about any adverse events and drug administration compliance. If the participant is tolerating the medication, the dose will be uptitrated from 30 mg daily to 45 mg daily.

Visit 4: Week 20

- Physical examination with vital signs
- Medication review
- Adverse events review
- Clinical laboratory tests (see Appendix I: Schedule of Assessments)
- Clinical assessments
- If the participant is on aspirin, other blood thinners, or anti-inflammatories (NSAIDS) they will be instructed to hold these medications for 5 days prior to the Week 32 muscle biopsy.

Visit 5: Week 32

- Physical examination with vital signs
- Medication review
- Adverse Events Review
- Clinical laboratory tests (see Appendix I: Schedule of Assessments)
- Diaries will be collected and participants will be given a new falls diary and medication diary
- Clinical assessments
- Muscle ultrasound
- Needle muscle biopsy
- 50 cc of blood will be collected and serum will be isolated and stored for laboratory study
- The Investigational Drug Pharmacy at Bayview will dispense study drug for a four-month period.

Visit 6: Week 48

- Physical examination with vital signs
- Medication review

- Adverse Events Review
- Clinical laboratory tests (see Appendix I: Schedule of Assessments)
- Diaries will be collected.
- Clinical assessments
- 50 cc of blood will be collected and serum will be isolated and stored for laboratory study

a. Study duration and number of study visits required of research participants.

   This is a 3 year study with subjects attending up to 6 study visits over a 12 month period.

b. Blinding, including justification for blinding or not blinding the trial, if applicable.
   NOT APPLICABLE.

c. Justification of why participants will not receive routine care or will have current therapy stopped.
   Routine care will not be stopped. There is no known effective treatment for IBM at this time.

d. Justification for inclusion of a placebo or non-treatment group.
   We will have a four month "lead-in" phase for all study participants. This will allow us to determine the natural history of the disease, including any change in mitochondrial biomarkers, prior to starting the study drug. No subjects will receive a placebo.

e. Definition of treatment failure or participant removal criteria.
   There is no known treatment for IBM and it is a slowly progressive disease, so the study drug is not necessarily a treatment failure if the participant clinically declines during the treatment period.

   A participant will be removed from the trial if they have an adverse reaction to pioglitazone.

   An investigator may decide to discontinue a subject from the study for safety reasons. A subject's participation in the study may be discontinued at any time. Reasons include, but are not limited to the following:
   -subject voluntarily withdraws consent
   -subject does not meet eligibility criteria
   -subject experiences an SAE assessed as possibly or probably related to study drug
   -subject is unwilling or unable to comply with the requirements of the protocol
   -subject becomes pregnant
   -subject is lost to follow-up/fails to return for visits

   When a subject does not complete the study, they will be asked to return for a final assessment.

f. Description of what happens to participants receiving therapy when study ends or if a participant's participation in the study ends prematurely.
   Participants will no longer be prescribed pioglitazone when the study ends or if a participant's participation ends prematurely.

5. Inclusion/Exclusion Criteria

The study population will consist of ambulatory patients with biopsy-confirmed or clinically defined s-IBM based on the 2010 criteria established by the European Neuromuscular Center. A diagnosis can be made based on defined clinical criteria or histopathology.

Eligibility Criteria:

1. Age ≥ 50 years
2. Diagnosis of sIBM based on the sIBM Diagnostic Criteria established by the 2010 European Neuromuscular Center. A diagnosis can be made based on defined clinical criteria or histopathology.
3. Must be able to ambulate at least 20 feet, with or without the use of an assistive device. Patients may not use another person, wall, or furniture for support.
4. Must be able to rise from a chair without support from another person or device.
5. Able to communicate with the investigator.
6. Ability to understand and willingness to sign a written informed consent document.
7. Premenopausal women must have a negative serum pregnancy test prior to dosing with study medication.
8. The effects of pioglitazone on the developing human fetus are unknown. For this reason and because TZD's are known to be teratogenic, women of child-bearing potential and men must agree to use adequate contraception (hormonal or barrier method of birth control; abstinence) prior to study entry and for the duration of study participation. Should a woman become pregnant or suspect she is pregnant while she or her partner is participating in this study, she should inform her treating physician immediately.
9. Must have viable quadriceps muscle suitable for biopsy as seen on ultrasound and interpreted by the investigator.

Exclusion Criteria:

1. A history of diabetes mellitus, or prior or concurrent treatment with any diabetes therapy (including but not limited to insulin, metformin, thiazolidinediones, pramlintide, alpha-glucosidase inhibitors, DPP-4 inhibitors, glucagon-like peptides (incretin-mimetics), meglitinides, sodium glucose transporter (SGLT) inhibitors).
2. Use of chronic immunosuppressive therapy including corticosteroids or IVIG within the past 6 months.
3. Use of Vitamin E supplements within the past 3 months
4. Creatine kinase (CK) > 15x the upper limit of normal
5. History of heart failure (New York Heart Association class II-IV) or symptoms of heart failure including lower extremity swelling, shortness of breath or chest pain.
6. Patients with any of the following hepatic conditions prior to study: (a) history of chronic liver or biliary disease, (b) total conjugated bilirubin greater than 1.5 times ULN range, unless in the context of Gilbert's syndrome, (c) alkaline phosphatase greater than 1.5 times the ULN range, (d) AST, ALT greater than 2.5 times the ULN if the elevation of AST or ALT, according to the investigator, is attributable to liver disease.
7. History of bladder cancer

8. Known osteoporosis, defined as a T score < 2.5 on most recent DEXA scan, or a history of a fragility fracture. This is because pioglitazone may increase the risk of osteoporosis in some patients.
9. Patients who are receiving any other investigational agents or have used an investigational drug within the past 6 months. Planned participation in another clinical trial during the duration of this study.
10. Alcohol or drug abuse within the past 6 months.
11. History of allergic reactions attributed to pioglitazone or compounds of similar chemical or biologic composition to pioglitazone.
12. Mal-nutrition or mal-absorptive syndrome defined as less than 0.8 g protein/kg/day or diseases known to cause malabsorption, including inflammatory bowel disease, celiac disease, short bowel syndrome or pancreatic insufficiency.
13. Any condition other than s-IBM that causes significant muscle pain, muscle weakness, muscle atrophy, or joint pain. This includes but is not limited to such neurologic and neuromuscular diseases as polymyositis or dermatomyositis, myasthenia gravis, amyotrophic lateral sclerosis, stroke, multiple sclerosis, epilepsy, muscular dystrophy, fibromyalgia, rheumatoid arthritis, spinal cord injury or degenerative disease of the spine. Osteoarthritis is not exclusionary unless it limits the patient's ability to comply with study tasks. Patients with a history of a hip or vertebral fracture within the past year or surgical hip or knee replacement within the past six months will be excluded.
14. Significant coagulopathy with platelet count < 100 or INR > 1.4. Use of daily aspirin that cannot be safely held for 5 days as determined by the study investigator (ie: prior stroke or heart attack)
15. Any medical condition that limits mobility. This includes but is not limited to symptomatic lung disease, Alzheimer's disease, Parkinson's disease, advanced organ failure including chronic kidney disease with GFR < 30 mL/min using the MDRD equation, uncontrolled endocrinologic disease including thyroid disease. Patients with hypothyroidism who have changed their dose of thyroid replacement therapy within the past 8 weeks are not eligible.
16. Planned procedure that would be anticipated to reduce mobility for over 1 week (for example, hip replacement surgery) during the planned 12 month study duration.
17. Patients receiving any medications or substances that are inhibitors or inducers of CYP2C8 are ineligible. These medications include but are not limited to Gemfibrozil, Rifampin, and warfarin. Pioglitazone is also metabolized by the CYP3A4 isoenzyme system, so strong inducers of CYP3A4 (e.g., rifampin, azoles, phenytoin, carbamazepine, phenobarbital, and St. John's wort, or strong inhibitors (e.g. clarithromycin and itraconazole)) should be used with caution but are not contraindicated. Because the lists of these agents are constantly changing, it is important to regularly consult a frequently-updated list such as http://medicine.iupui.edu/clinpharm/ddis/; medical reference texts such as the Physicians' Desk Reference may also provide this information. As part of the enrollment/informed consent procedures, the patient will be counseled on the risk of interactions with other agents, and what to do if new medications need to be prescribed or if the patient is considering a new over-the-counter medicine or herbal product. Uncontrolled intercurrent illness including, but not limited to, ongoing or active infection, symptomatic congestive heart failure, unstable angina pectoris, cardiac arrhythmia, or psychiatric illness/social situations that would limit compliance with study requirements.
18. Pregnant women are excluded from this study because pioglitazone is an agent with the potential for teratogenic or abortifacient effects. Because there is an unknown but potential risk

for adverse events in nursing infants secondary to treatment of the mother with pioglitazone, breastfeeding should be discontinued if the mother is treated with pioglitazone.

19. History of cancer less than five years prior, other than local basal or squamous cell cancer.
20. Patient has any medical condition or laboratory finding during screening, which, in the investigator's opinion may interfere with participation, confound the results, or pose any additional risk to the patient.

6. Drugs/ Substances/ Devices

a. The rationale for choosing the drug and dose or for choosing the device to be used.

Drug name/synonyms: pioglitazone (Actos®)
Active ingredient: pioglitazone hydrochloride
Inactive ingredients: lactose monohydrate NF, hydroxypropylcellulose NF, carboxymethylcellulose calcium NF, and magnesium stearate NF.
Dosage: 15 mg tablets, total dose of 45 mg (3 tablets) daily by oral administration.

Pioglitazone can be taken with or without food.
Pioglitazone tablets should be stored in the original container at room temperature away from moisture, heat or light.

Pioglitazone is a generic drug of the thiazolidinedione (TZD) class that is an insulin sensitizing agent approved for the treatment of type 2 diabetes. The pharmacologic target of pioglitazone is the nuclear receptor peroxisome proliferator-activated receptor gamma (PPARγ), which modulates the expression of multiple genes involved in insulin signaling and glucose metabolism in peripheral tissues. Pioglitazone increases expression of activated AMPK and PGC-1α in human skeletal muscle of patients with diabetes [12], resulting in increased mitochondrial biogenesis and improved exercise capacity and mitochondrial function [13,14]. We hypothesize that pioglitazone will increase mitochondrial biogenesis in skeletal muscle from patients with IBM and therefore improve muscle function.

Pioglitazone has been safely administered to normoglycemic elderly patients in clinical trials for Alzheimer's disease [15] and it has also been studied in non-alcoholic steatohepatitis [16], among other diseases. We plan to start pioglitazone at a dose of 30 mg daily and uptitrate by 15 mg after 2 weeks for a goal dose of 45 mg daily, which is consistent with the FDA label and was the starting dose used in the non-alcoholic steatohepatitis trial [16].

The pharmacokinetics of pioglitazone is well established. Pioglitazone is well absorbed after oral administration, and peak concentrations are achieved approximately 1.5 hours after oral administration. The half-life is around 9 hours, and steady-state is achieved within 7 days after starting pioglitazone. Pioglitazone is metabolized predominately by the liver and inactive metabolites are excreted in the faeces [17]. The dose does not need to be adjusted in patients with kidney disease or hepatic failure [18]. Pioglitazone is metabolized by CYP2C8 and CYP3A4 [19], and therefore CYP2C8 or CYP3A4 inducers or inhibitors should be avoided or used with caution.

Lidocaine with epinephrine will be used as a local anesthetic to the biopsy sites prior to the biopsy procedure. The drug used in the study will be the same drug provided for the entire

Johns Hopkins Hospital. There will not be a separate batch of this drug for the sole purposes of this study as use of the drug is unlikely to interfere with study results.

b. Justification and safety information if FDA approved drugs will be administered for non-FDA approved indications or if doses or routes of administration or participant populations are changed.

For the purposes of this investigation, pioglitazone will be used in the same dose, duration, route of administration as described in the current labeling. Pioglitazone is currently approved for type 2 diabetes only, although it has been studied in other populations including polycystic ovarian disease, Alzheimer's disease, and non-alcoholic steatohepatitis as discussed above.

Pioglitazone is generally well tolerated. Common side-effects include headaches, increased upper respiratory tract infections, weight gain and fluid retention. In contrast to many other antidiabetic agents such as insulin and sulfonylureas, pioglitazone does not by itself cause hypoglycemia in nondiabetic patients. Pioglitazone is contraindicated in patients with known Class III or IV heart failure, because fluid retention induced by the drug can cause or exacerbate heart failure. There are some post-marketing reports of liver failure in patients treated with pioglitazone, although these reports were never able to confirm causality. Patients with elevated liver function tests can still be treated with pioglitazone, but caution is advised. There is also an increased incidence of fractures in female patients treated with pioglitazone. Lastly, there is possibly an increased risk of bladder cancer in pioglitazone users, although the number of cases of bladder cancer were too small to establish causality.

Because of available safety data in multiple different diseases, we believe the use of pioglitazone in sIBM will be safe as well as justified to study.

Additionally, pioglitazone is lawfully marketed in the United States. The investigation is not intended to be reported to the FDA as a well-controlled study in support of a new indication and there is no intent to use it to support any other significant change in the labeling of the drug. Pioglitazone is a prescription drug, but the current investigation is not intended to support a significant change in the advertising of the drug. The investigation does not involve a route of administration, dose, patient population, or other factor that significantly increases the risk (or decreases the acceptability of the risk) associated with the use of the drug product.

c. Justification and safety information if non-FDA approved drugs without an IND will be administered. NOT APPLICABLE.

7. Study Statistics

Primary outcome variable:
The purpose of this study is to test the hypothesis that pioglitazone can ameliorate mitochondrial deficits in sIBM. We will use RNA sequencing and Ingenuity pathway analysis of muscle tissue to quantify changes in expression of genes involved in oxidative phosphorylation and mitochondrial function before and after treatment with pioglitazone. We will compare the mean change in mRNA expression on treatment (week 16 to week 32) to the mean change in mitochondrial function and mRNA expression off treatment (baseline to week 16) using a paired t-test with appropriate transformations if needed.

Secondary and exploratory outcome variables:

We will measure the metabolome, mitochondrial respiration and glycolysis. We will compare the mean change in mitochondrial function and metabolic markers on treatment (week 16 to week 32) to the mean change in mitochondrial function, metabolic markers and mRNA expression off treatment (baseline to week 16) using a paired t-test with appropriate transformations if needed. Descriptive statistics will be used to describe clinical changes observed on pioglitazone therapy. In particular, we will describe any clinical changes in muscle strength, functional measures, IBM-FRS, number of falls, and changes on ultrasound that occur during pioglitazone therapy (weeks 32 and 48) as compared to week 16 when pioglitazone therapy is initiated. Additionally, we will describe the rate of change of these clinical endpoints over the 16 week study lead-in before therapy is started to the 32 week period after therapy is initiated. We will present these changes visually (scatterplots, graphs) and descriptively.

For secondary objective #9, we will use the Student's t-test to compare mitochondrial function and mitochondrial RNA expression in affected vs unaffected muscle before treatment is initiated.

Safety and tolerability of pioglitazone will be described using incidence of AEs reported and observed, number of patients discontinuing treatment due to an AE or SAE, and changes from baseline in vital signs, weight, clinical laboratory test results, and physical examination findings over time.

<u>Sample Size Considerations</u>
This is an exploratory study with an empirical sample size considered to be sufficient to formulate initial hypotheses. This study will allow us to quantify the effect size and standard deviation, which will be important for future studies of pioglitazone in IBM.

<u>Population for Analysis</u>
All participants enrolled and receiving therapy will be included in the safety analysis. All participants who received at least 16 weeks of treatment and receive an on-treatment biopsy will be included in the efficacy population and efficacy analysis.
Descriptive statistics such as number of observations, mean, median, standard deviation, standard error, minimum and maximum will be presented by groups for continuous variables (such as age, weight, etc.). Other descriptive statistics such as counts, proportions, and/or percentages will be presented by group to summarize discrete variables (such as race, sex, etc.)

<u>Demographic and baseline characteristics</u>
The number of participants completed and discontinued early from study and the reasons for the discontinuation will be summarized. Demographic and baseline characteristics will also be summarized. Variables include race, age, sex, and selected clinical variables recorded prior to initiation of study drug. Study drug administration will be summarized in terms of number of days of dosing and reasons for final discontinuation of study drug.

8. Risks

Risks with pioglitazone have been identified based on clinical findings from previous clinical trials. Common side-effects include headaches, increased upper respiratory tract infections, weight gain and fluid retention. In contrast to many other antidiabetic agents such as insulin and sulfonylureas, pioglitazone does not by itself cause hypoglycemia in nondiabetic patients. Pioglitazone is contraindicated in patients with known Class III or IV heart failure, because fluid retention induced by the drug can cause or exacerbate heart failure. There are some post-marketing reports of liver failure in patients treated with pioglitazone, although these reports were never able to confirm

causality. Patients with elevated liver function tests can still be treated with pioglitazone, but caution is advised. There is also an increased incidence of fractures in female patients treated with pioglitazone. Lastly, there is possibly an increased risk of bladder cancer in pioglitazone users, although the number of cases of bladder cancer were too small to establish causality.

Risks also include potential bleeding, discomfort, and a slight risk of infection from the muscle biopsies. As with any cut in the skin, a scar will develop, although its appearance will likely fade over time. Local anesthesia will be used to minimize discomfort. Subjects will be instructed on optimum wound management to minimize scarring and infection risk.

Local anesthesia may cause minor discomfort (a burning sensation), redness, and/or swelling.

Risks associated with blood sampling include discomfort, bleeding, or bruising where the needle enters the body. It may also result in fainting. There is a small risk of infection.

There are no anticipated risks or discomforts associated with the physical exams, vital sign checks, muscle testing, questionnaires, and clinical assessments/grading.

There is a potential risk to confidentiality. Most participant related data will be stored in our institution's electronic medical record and paper files. If members from the IRB, NIH, or FDA need to inspect trial records, they will be released. Hence, absolute confidentiality cannot be guaranteed. Every effort will be made to keep the participants' study information and records safe by following university and HIPAA policy for handling PHI. Participants will be assigned a unique identifier number which will be used to identify the patient's information as well as tissue samples during the study. The results of this research project may be presented at meetings or in publications; however, participants' identity will not be disclosed.

There is a slight financial risk to the participants in the event that the above complications occur requiring additional medical care.

Data and Safety Monitoring: Any clinical and incidental findings determined by the Investigator to be important and/or unusual will be referred to as an adverse event (AE). The Investigators will use their discretion to remove participants from the study and will follow-up the case to assess the cause, effect, and severity of any AE, and findings will be documented on an AE form. Risk events, problems, and deviations will be immediately reported by the PI to the IRB.

If an ongoing AE changes in its intensity or in its perceived relationship to study drug, a new AE entry for the event will be completed. Adverse events will be followed to resolution or stabilization, or reported as SAEs if they become serious. Follow-up is also required for AEs that cause interruption or discontinuation of study drug, or those that are present at the end of study participation. Participants with AEs at study completion will receive post-treatment follow-up as appropriate.

All identified AEs will be recorded and described on the appropriate Case Report Form (CRF). Non-serious adverse events will be logged and reported to the IRB on an annual basis. All serious adverse events, regardless of causality will be reported to the IRB per the JHMIRB reporting requirements. Prompt reporting to the JHMIRB of unanticipated problems and SAEs will occur as soon as possible after the PI learns of the event, but in all cases within 10 working days with the exception of death of a JHM participant. In this instance, reporting requirements defined in Policy 103.6(b)(i) will be followed.

An adverse event (AE) is any untoward medical occurrence (i.e., any unfavorable and unintended sign [including abnormal laboratory findings], symptom or disease) in a subject or clinical investigation subject *after providing written informed consent* for participation in the study. Therefore, an AE may or may not be temporally or causally associated with the use of a medicinal (investigational) product.

Adverse events should be sought by non-directive questioning of the subject at each visit during the study. Adverse events also may be detected when they are volunteered by the subject during or between visits or through physical examination, laboratory test, or other assessments. Abnormal laboratory values or test results constitute adverse events only if they fulfill at least one of the following criteria:

- they induce clinical signs or symptoms,
- they are considered clinically significant,
- they require therapy.

Clinically significant abnormal laboratory values or test results should be identified through a review of values outside of normal ranges/clinically notable ranges, significant changes from baseline or the previous visit, or values which are considered to be non-typical in subject with underlying disease. Investigators have the responsibility for managing the safety of individual subject and identifying adverse events. Adverse events should be recorded in the Adverse Events CRF under the signs, symptoms or diagnosis associated with them accompanied by the following information.

- the severity grade:
  - mild: usually transient in nature and generally not interfering with normal activities
  - moderate: sufficiently discomforting to interfere with normal activities
  - severe: prevents normal activities
- its relationship to the study treatment (no/yes)
- its duration (start and end dates). If the event is ongoing and does not resolve it should be reported
- whether it constitutes a serious adverse event (SAE)
- action taken regarding study treatment
- whether other medication or therapies have been taken (concomitant medication/non-drug therapy)
- its outcome (not recovered/not resolved; recovered/resolved; recovering/resolving, recovered/resolved with sequelae; fatal; or unknown)

An SAE is any adverse event (appearance of (or worsening of any pre-existing) undesirable sign(s), symptom(s) or medical conditions(s) which meets any one of the following criteria

- is fatal or life-threatening
- results in persistent or significant disability/incapacity
- constitutes a congenital anomaly/birth defect
- requires inpatient hospitalization or prolongation of existing hospitalization, unless hospitalization is for:
- routine treatment or monitoring of the studied indication, not associated with any deterioration in condition
- elective or pre-planned treatment for a pre-existing condition that is unrelated to the indication under study and has not worsened since signing the informed consent

- treatment on an emergency outpatient basis for an event not fulfilling any of the definitions of a SAE given above and not resulting in hospital admission
- social reasons in the absence of any deterioration in the subject's general condition
- is medically significant, i.e. defined as an event that jeopardizes the subject or may require medical or surgical intervention to prevent one of the outcomes listed above.

Deidentified individual participant-level data will be shared with the Vivli repository. Data will only be made available upon request, which will be reviewed by an approving entity. If approved, the researcher must sign a Data Use Agreement (DUA) prior to accessing the data.

9. Benefits

There are no direct benefits from participation in this study. If the results are favorable, use of pioglitazone could improve muscle strength and function in sIBM.

10. Payment and Remuneration

Participants will receive $40 per study visit. Therefore, a maximum of $240 is payable to subjects upon completion of all 6 study visits. Parking validation will also be provided to cover the cost of parking at the Bayview Medical Campus.

11. Costs

Study participants will not be responsible for any study related costs. Study costs will be funded by internal grants within the Division of Rheumatology: the Ira T. Fine Discovery Fund and the Jerome L. Greene Foundation Discovery Fund. We do not anticipate receiving federal funding for this study.

References

1. Needham, M. *et al.* Prevalence of sporadic inclusion body myositis and factors contributing to delayed diagnosis. *J. Clin. Neurosci.* **15,** 1350–3 (2008).
2. Paltiel, A. D. *et al.* Demographic and clinical features of inclusion body myositis in North America. *Muscle Nerve* **52,** 527–33 (2015).
3. Rifai, Z., Welle, S., Kamp, C. & Thornton, C. A. Ragged red fibers in normal aging and inflammatory myopathy. *Ann. Neurol.* **37,** 24–9 (1995).
4. Oldfors, A. *et al.* Mitochondrial abnormalities in inclusion-body myositis. *Neurology* **66,** S49-55 (2006).
5. Johnson, L. G. *et al.* Improvement in aerobic capacity after an exercise program in sporadic inclusion body myositis. *J. Clin. Neuromuscul. Dis.* **10,** 178–84 (2009).
6. Arnardottir, S., Alexanderson, H., Lundberg, I. E. & Borg, K. Sporadic inclusion body myositis: pilot study on the effects of a home exercise program on muscle function, histopathology and inflammatory reaction. *J. Rehabil. Med.* **35,** 31–5 (2003).
7. Felice, K. J., Relva, G. M. & Conway, S. R. Further observations on forearm flexor weakness in inclusion body myositis. *Muscle Nerve* **21,** 659–61 (1998).
8. Needham, M. *et al.* Sporadic inclusion body myositis: phenotypic variability and influence of HLA-DR3 in a cohort of 57 Australian cases. *J. Neurol. Neurosurg. Psychiatry* **79,** 1056–60 (2008).
9. Holloszy, J. O. & Booth, F. W. Biochemical adaptations to endurance exercise in muscle. *Annu. Rev. Physiol.* **38,** 273–91 (1976).

10. Safdar, A. *et al.* Exercise increases mitochondrial PGC-1alpha content and promotes nuclear-mitochondrial cross-talk to coordinate mitochondrial biogenesis. *J. Biol. Chem.* **286,** 10605–17 (2011).

11. Shabrokh, E. *et al.* Mitochondrial Dysregulation in Skeletal Muscle from Patients Diagnosed with Alzheimer's Disease and Sporadic Inclusion Body Myositis. *Open J. Mol. Integr. Physiol.* **4,** 11–19 (2014).

12. Coletta, D. K. *et al.* Pioglitazone stimulates AMP-activated protein kinase signalling and increases the expression of genes involved in adiponectin signalling, mitochondrial function and fat oxidation in human skeletal muscle in vivo: a randomised trial. *Diabetologia* **52,** 723–32 (2009).

13. Takada, S. *et al.* Pioglitazone ameliorates the lowered exercise capacity and impaired mitochondrial function of the skeletal muscle in type 2 diabetic mice. *Eur. J. Pharmacol.* **740,** 690–696 (2014).

14. Pagel-Langenickel, I. *et al.* PGC-1alpha integrates insulin signaling, mitochondrial regulation, and bioenergetic function in skeletal muscle. *J. Biol. Chem.* **283,** 22464–72 (2008).

15. Geldmacher, D. S., Fritsch, T., McClendon, M. J. & Landreth, G. A randomized pilot clinical trial of the safety of pioglitazone in treatment of patients with Alzheimer disease. *Arch. Neurol.* **68,** 45–50 (2011).

16. Belfort, R. *et al.* A Placebo-Controlled Trial of Pioglitazone in Subjects with Nonalcoholic Steatohepatitis. *N Engl J Med* **22355,** 2297–307 (2006).

17. Eckland, D. A. & Danhof, M. Clinical pharmacokinetics of pioglitazone. *Exp. Clin. Endocrinol. & Diabetes* **108,** 234–242 (2000).

18. Budde, K. *et al.* The pharmacokinetics of pioglitazone in patients with impaired renal function. *Br. J. Clin. Pharmacol.* **55,** 368–74 (2003).

19. Jaakkola, T., Laitila, J., Neuvonen, P. J. & Backman, J. T. Pioglitazone is Metabolised by CYP2C8 and CYP3A4 in vitro: Potential for Interactions with CYP2C8 Inhibitors. *Basic Clin. Pharmacol. Toxicol.* **99,** 44–51 (2006).

20. Bogacka, I., Xie, H., Bray, G. A. & Smith, S. R. Pioglitazone induces mitochondrial biogenesis in human subcutaneous adipose tissue in vivo. *Diabetes* **54,** 1392–9 (2005).

21. Wang, H., Hiatt, W. R., Barstow, T. J. & Brass, E. P. Relationships between muscle mitochondrial DNA content, mitochondrial enzyme activity and oxidative capacity in man: alterations with disease. *Eur. J. Appl. Physiol. Occup. Physiol.* **80,** 22–7 (1999).

22. Mihaylova, M. M. & Shaw, R. J. The AMPK signalling pathway coordinates cell growth, autophagy and metabolism. *Nat. Cell Biol.* **13,** 1016–23 (2011).

23. Bergeron, R. *et al.* Chronic activation of AMP kinase results in NRF-1 activation and mitochondrial biogenesis. *Am. J. Physiol. Endocrinol. Metab.* **281,** E1340-6 (2001).

24. Federation of American Societies for Experimental Biology., I., Mantell, B. & Sack, M. N. *Federation proceedings. The FASEB Journal* **20,** (Federation of American Societies for Experimental Biology, 2006).

25. Jové, M. *et al.* Impaired expression of NADH dehydrogenase subunit 1 and PPARgamma coactivator-1 in skeletal muscle of ZDF rats: restoration by troglitazone. *J. Lipid Res.* **45,** 113–23 (2004).

26. Catoire, M. *et al.* Pronounced Effects of Acute Endurance Exercise on Gene Expression in Resting and Exercising Human Skeletal Muscle. *PLoS One* **7,** e51066 (2012).

27. Noto, Y.-I. *et al.* Contrasting echogenicity in flexor digitorum profundus-flexor carpi ulnaris: A diagnostic ultrasound pattern in sporadic inclusion body myositis. *Muscle Nerve* **49,** 745–748 (2014).

28. Adler, R. S., Garolfalo, G., Paget, S. & Kagen, L. Muscle sonography in six patients with hereditary inclusion body myopathy. *Skeletal Radiol.* **37,** 43–48 (2007).

29. Nodera, H. *et al.* Intramuscular dissociation of echogenicity in the triceps surae characterizes sporadic

inclusion body myositis. *Eur. J. Neurol.* **23,** 588–96 (2016).

30. Bohannon, R. W. Reference values for the timed up and go test: a descriptive meta-analysis. *J. Geriatr. Phys. Ther.* **29,** 64–8 (2006).

31. Shumway-Cook, A., Brauer, S. & Woollacott, M. Predicting the probability for falls in community-dwelling older adults using the Timed Up & Go Test. *Phys. Ther.* **80,** 896–903 (2000).

32. Kristensen, M. T., Foss, N. B. & Kehlet, H. Timed "up & go" test as a predictor of falls within 6 months after hip fracture surgery. *Phys. Ther.* **87,** 24–30 (2007).

Appendix I: Schedule of Assessments

	Screening Period	Baseline / Lead-in Period	Pioglitazone: 30 mg oral daily, escalating to 45 mg oral daily				
	Day -30 to 0	Week 0	Week 16	Week 18	Week 20	Week 32	Week 48
	Visit 1	Visit 2*	Visit 3*	phone call	Visit 4	Visit 5*	Visit 6
Informed consent	X						
Demographics	X						
Medical history	X						
Concurrent meds	X	X	X	X	X	X	X
Physical exam	X	X	X		X	X	X
Vital signs	X	X	X		X	X	X
CBC	X		X		X	X	X
CMP	X						
Creatinine			X		X	X	X
Fasting glucose			X		X	X	X
HbA1c	X						
pro-BNP	X				X	X	X
Liver function tests (AST, ALT, Alk phos, bilirubin)			X		X	X	X
INR, PTT	X		X			X	
CK	X		X			X	X
B-HCG (only women of childbearing potential)	X						
Research blood (up to 50 CCs)		X	X			X	X
Muscle ultrasound	X	X	X			X	X
Muscle needle biopsy		X	X			X	
Manual Muscle Testing		X	X			X	X
Quantitative Dynamometry		X	X			X	X
m-TUG		X	X			X	X
Fi-2		X	X			X	X
6-minute walk test		X	X			X	X
IBM-FRS		X	X			X	X
Falls Diary Review		X	X		X	X	X
Medication Diary Review			X	X	X	X	X
Adverse event assessment	X	X	X	X	X	X	X

*For scheduling purposes, study procedures for Visit 2/Week 0, Visit 3/Week 16, Visit 5/Week 32 may be conducted over 2 consecutive days.
